# Extra Virgin Olive Oil Quality as Affected by Yeast Species Occurring in the Extraction Process

**DOI:** 10.3390/foods8100457

**Published:** 2019-10-07

**Authors:** Simona Guerrini, Eleonora Mari, Damiano Barbato, Lisa Granchi

**Affiliations:** 1 FoodMicroTeam s.r.l., Academic Spin-Off of the University of Florence, via Santo Spirito, 14-50125 Florence, Italy; simona@foodmicroteam.it; 2 Department of Agriculture, Food, Environment and Forestry (DAGRI), P.le delle Cascine, 24-50144 Florence, Italy; eleonora.mari@unifi.it (E.M.); damiano.barbato@unifi.it (D.B.)

**Keywords:** yeast microbiota, extra virgin olive oil, *Nakazawaea molendini-olei*, *Nakazawaea wickerhamii*, *Yamadazyma terventina*, yeast enzymatic activities, volatile compounds, sensory analysis

## Abstract

In extra virgin olive oil (EVOO) extraction process, the occurrence of yeasts that could affect the quality of olive oil was demonstrated. Therefore, in this work, at first, the yeasts occurring during different extractive processes carried out in a Tuscany oil mill, at the beginning, in the middle, and the end of the harvesting in the same crop season, were quantified. Then, possible effects on quality of EVOO caused by the predominant yeast species, possessing specific enzymatic activities, were evaluated. Yeast concentrations were higher in extraction processes at the end of the harvesting. Twelve yeast species showing different isolation frequencies during olive oil extractive process and according to the harvesting date were identified by molecular methods. The yeast species dominating olive oil samples from decanter displayed enzymatic activities, potentially affecting EVOO quality according to zymogram analysis. HS-SPME-GC-MS analysis of the volatile compounds in commercial EVOO, inoculated with three yeast species (*Nakazawaea molendini-olei*, *Nakazawaea* *wickerhamii*, *Yamadazyma terventina*), pointed out significant differences depending on the strain inoculated. In conclusion, during the olive oil extractive processes, some yeast species colonize the extraction plant and may influence the chemical and sensory characteristics of EVOO depending on the cell concentrations and their enzymatic capabilities.

## 1. Introduction

Extra virgin olive oil (EVOO) is not just a product obtained from the fruit of the olive tree by mechanical extraction, but rather the result of complex changes in fruit components. Because of these changes, chemical compounds affecting the qualitative characteristics for sensory acceptability of extra virgin olive oil [1] may be produced. Pleasant sensory notes, characterizing extra virgin olive oil, are mainly originated from aldehydes, esters, alcohols, and ketones, which are responsible for oil sensory attributes such as “green” and “fruity” [2,3,4,5,6]. Nevertheless, several phenomena can alter the initial pleasant flavor, giving rise to unpleasant sensory notes, classified, according to the current olive oil regulations (EU Reg. 1348/2013), into four groups: “fusty”, “musty”, “winey–vinegary”, and “rancid”. Microorganisms associated with the olives may affect oil quality according to their metabolic activities. Indeed, as reported by Vichi et al. [7,8,9], oils from microbiologically contaminated olives exhibited a lower quality level and influences of olive microbiota on oil characteristics were greater than the effects exerted by malaxation time and temperature. Guerrini et al. [10] showed that sensory defects and specific volatile compounds (i.e., 2-butanone, butyric acid, 2-heptanol, octanoic acid, 1-octen-3-ol) were correlated to both yeast and mould concentrations detected in extracted and filtered oils. Yeasts and moulds are present in extracted oil because, during olive crushing, microorganisms of olives pass on into oil through both solid particles of olive fruit and micro-drops of vegetation water [11,12,13]. Some yeast species occurring in newly unfiltered oil can remain viable and metabolically active during the conservation period and, according to their metabolic capabilities, can either improve or worsen the oil quality [9]. Enzymatic activities of yeasts isolated from either olives or olive oil have been reported to include β-glucosidase, β-glucanase, polyphenoloxidases, peroxidase, lipase and cellulase activities [11,14,15,16,17]. Enzymes such as ß-glucosidase are known to improve oil quality by increasing phenolic compound extractability, while others such as lipase, polyphenoloxidases, and peroxidase are known to cause detrimental effects [5,18,19,20]. Recent studies [17,21] demonstrated that the presence of some yeast species might be responsible for olive oil sensory decay during storage. In particular, laboratory experiments showed the presence of defects in olive oil treated with specific yeast strains of *Candida adriatica*, *Nakazawaea wickerhamii,* and *Candida diddensiae*, while other olive oil samples treated with other *Candida diddensiae* strains were defect-free after four months of storage [22,23]. By the way, yeasts belonging to various genera were isolated in commercial extra virgin olive oil (*Candida*, *Nakazawaea*, *Williopsis*, *Ogataea*, *Yamadazyma* and *Saccharomyces*) [11,12,13,14,24,25,26,27].

Despite these evidence regarding the presence of viable yeasts in oil and their potential impact on olive oil quality, only few studies have investigated the yeast species occurring in the different phases of the olive oil extraction process and their effects on the oil quality [16,23,28]. In particular, a recent study of Mari et al. [28]. Mari et al. showed that the yeast populations occurring in olive oil extraction processes are numerically significant and originate principally from the yeasts colonizing the oil extractive plants. In fact, this study showed that only three of the eleven dominant yeast species detected on the washed olives were also found in extracted oil at significant isolation frequencies (*Candida adriatica*, *Nakazawaea molendini-olei*, and *Nakazawaea wickerhamii*). On the contrary, some yeast species showed significant isolation frequencies only in extracted oil (*Yamadazyma terventina*), or in kneaded pastes and pomaces (*Zygotorulaspora mrakii*). The occurrence of different yeast species according to the source of isolation (pastes, extracted oil or pomaces) suggests a contamination of the plant during oil extraction that select specific yeast species [28]. Ciafardini et al. [29] found a lower species diversity based on the origin of isolation. These Authors found six different yeast species (*Kluyveromyces marxianus*, *Candida oleophila*, *Candida diddensiae*, *Candida norvegica*, *Wickerhamomyces anomalus* and *Debaryomyces hansenii*). Except from *K. marxianus* that was found only in the wash water and *W. anomalus* that was found only in the six-month stored olive oil, all the other species occurred in the wash water and in the kneaded paste as well as in the newly produced olive oil. Anyway, a selected microbiota, when numerically significant, could affect olive oil quality in different ways, based on the specific metabolic capabilities of each yeast species or even strain. Therefore, the aim of this study was to assess whether the yeast microbiota occurring in olive oil extraction process affects the quality of extra virgin olive oil and, in particular, the volatile compounds content

For this purpose, at first, yeast species present during different extractive processes carried out in the same crop season as well as the chemical and sensory characteristics of the resulting olive oils were investigated. Then, some isolates belonging to the yeast species present at higher frequency in the process and possessing some enzymatic activities were inoculated into a commercial olive oil in order to assess their effective effects on extra-virgin olive oil quality.

## 2. Materials and Methods

### 2.1. Sampling throughout Olive Oil Extraction Processes

During the same crop season, 14 batches of approx. 200 kg olives, form Frantoio, Moraiolo, and mixed cultivars, were processed in a Tuscany oil mill (Azienda Agricola Buonamici s.r.l., Fiesole, Florence, Italy). Olives were collected and processed, within 4 h of harvesting, in three different harvest time at ten-day intervals: 6 at the beginning (HD1a, HD1b, HD1c, HD1d, HD1e and HD1f), 5 in the middle (HD2a, HD2b, HD2c, HD2d and HD2e) and 3 at the end (HD3a, HD3b and HD3c). Plant for oil extraction (TEM, Florence, Italy) consisted of a cleaning and water washing system, an olive grinding cutter crusher (mod. FR350), a controlled-temperature vertical axis malaxation equipment (500 kg capacity) (mod. V500), a “decanter” (two-step mod. D1500) with 1500 kg/h maximum capacity and a cardboard filter press (15 μm cut-off). Plastic residue or “alperujo” from decanter was subjected to separation by centrifugation of stone fragments to obtain destoned pomace. Olives were crushed at 2500 rpm (crusher holes 6.5 mm in diameter); malaxation was carried out at half capacity under vacuum (residual pressure of 20 kPa) at 22 ± 1 °C for a mean time of 15 min. Decanter worked with a screw conveyor rotating at a slower speed than that of the bowl. Samples were collected in double in several steps of extraction processes (washed olives, crushed and kneaded pastes, oils from decanter, pomaces), for microbial, chemical and sensory analyses (filtered oils).

### 2.2. Microbiological Analysis: Enumeration of Yeast Populations

Yeasts were quantified on MYPG agar (malt extract 5 g/L, yeast extract 3 g/L; beef extract 5 g/L, D-glucose 10 g/L) containing sodium propionate (2 g/L) and chloramphenicol (30 mg/mL) in order to inhibit growth of moulds and bacteria, respectively. The samples of olives, pastes and pomaces were plated after decimal dilutions (10 g in 90 mL of physiological saline solution: NaCl, 0.86 g/L homogenized in a Stomacher^®^ 400 (International Pbi, S.P.A., Milano, Italy) for 1 min.

Oil samples from decanter were plated after decimal dilutions (10 mL in 90 mL of physiological saline solution) or by filtration of 10 mL and subsequent washings with physiological solution through 0.45-µm cellulose membranes (Pall Corporation). Yeast colonies were counted after incubation for 48–72 h at 30 °C under aerobic conditions.

### 2.3. Chemical and Sensory Analyses of Olive Oil

The volatile compounds content was determined according to the literature [30], using solid phase microextraction of the headspace, coupled with a gas chromatograph with a mass spectrometer as a detector (HS-SPME-GC-MS technique). Analysis was performed using the Trace CG instrument combined with a Trace DSQ Thermo Finnigan instrument (Fisher Scientific SAS, Illkirch, France). Quantitative analysis was performed using 4-methyl-2-pentanol as an internal standard. Results were expressed as mg of aromatic compound per Kg of oil.

Acidity (expressed as percentage of oleic acid), peroxide value (meq O_2_/Kg) and total phenolic concentration (expressed as mg/Kg of gallic acid) were measured according to EU official method (EC Reg. 1989/2003) [31].

Sensory evaluation of olive oil was performed by a panel test according to the EU official method (EU Reg. 1348/2013) [32]. Samples were analyzed by a panel of professional tasters (8 tasters and a panel leader) recognized by MIPAAF (Ministry of Agricultural Policies, Food and Forestry) since 2002. Intensity of sensory defects and “fruity”, “bitter” and “pungent” attributes was assessed and expressed as the median of tasters score on a scale ranging from 0 to 10. 

### 2.4. Molecular Identification of Yeasts

From plates of each sample (washed olives, crushed and kneaded pastes, oils from decanter, pomaces) containing about 300 colonies, 20 colonies were purified and yeast isolates were stored in liquid medium containing 50% (*v*/*v*) glycerol at −80 °C until further use. Molecular identification of yeast isolates was performed by Randomly Amplified Polymorphic DNA (RAPD) analysis using the primer M13 (5’-GAGGGTGGCGGTTCT-3’) or D1/D2 26S rRNA gene sequencing analysis as reported by Mari et al. [24]. Relative frequencies of isolation used to represent yeast species density according to the isolation source, were calculated as the number of isolates belonging to each species divided by the total number of isolates and expressed in percentage.

### 2.5. Zymogram Screening for Yeast Enzymatic Activities

72 yeast isolates, belonging to the yeast species most frequently found in oil samples from decanter, were screened for enzymatic activities of potential interest in terms of olive oil quality as reported by Romo-Sánchez et al. [16]. The enzymatic activities screened were cellulase, polygalacturonase, ß-glucosidase, peroxidase, and lipase. The substrates used were, respectively, carboxymethylcellulose (CMC), polygalacturonic acid, cellobiose, H_2_O_2_, and CaCl_2_/Tween 80 (all purchased to Sigma Aldrich). Each isolate was grown in YPD (yeast extract 10 g/L; peptone 20g/L, D-glucose 20 g/L) broth at 30 °C for 24 h. To check for lipase activity, cultures were inoculated into 0.1% olive oil integrated with 0.01% Tween 80 broth. Cultures for checking cellulase and ß-glucosidase activity were then grown in a yeast nitrogen base (YNB) broth at 30 °C for 6 h under shaking conditions (100 rpm) for consumption of residual carbon source. Aliquots of 5 µL at 10^6^ CFU/mL were spotted on agar plates containing YP (yeast extract 10 g/L; peptone 20 g/L, agar 15 g/L) and 1% of each specific substrate as single carbon source. All plates were incubated at 28 °C for 3 days, except for lipase activity for 7 days. The activity was detected for clear halo for polygalactunorase, according to Fernández González et al., [33], appearance of white precipitation areas for lipase, [34] or growth for cellulase and ß-glucosidase [35]. Peroxidase activity was assessed by oxygen bubble production from H_2_O_2_.

### 2.6. Yeast Inoculation into Commercial EVOO

Some isolates belonging to the dominant yeast species (*C. adriatica*, *N. wickeramii*, *N. molendini*-*olei*, *Y. terventina*) and possessing some enzymatic activities were inoculated into a commercial filtered extra-virgin olive oil (EVOO). Each pure isolate was grown in YPD medium until the early stationary phase and then yeast cells were inoculated in order to have a final concentration of 10^6^ cell/mL. The inoculated oil and samples without inoculum as control were placed in sterile glass tubes and bottles, in the dark at a temperature of 15 °C for 180 days until microbial and chemical analysis.

### 2.7. Statistical Analysis

Microbiological determinations, performed in duplicate, were elaborated according to nonparametric ANOVA followed by Bonferroni Test. Differences were reported at a significance level of *p* < 0.05. Principal Component Analysis (PCA) was used to classify samples. Correlation studies between yeast concentration and the volatile compounds content of oil samples were carried out by calculating both Pearson and Spearman rank correlation coefficients (significance level: α = 0.05). All the statistical analyses were performed by Statistica 7.0 software package (Stasoft GmbH, Hamburg, Germany).

## 3. Results

### 3.1. Yeast Concentrations Occurring in Different Extractive Olive Oil Processes

**Figure 1:**
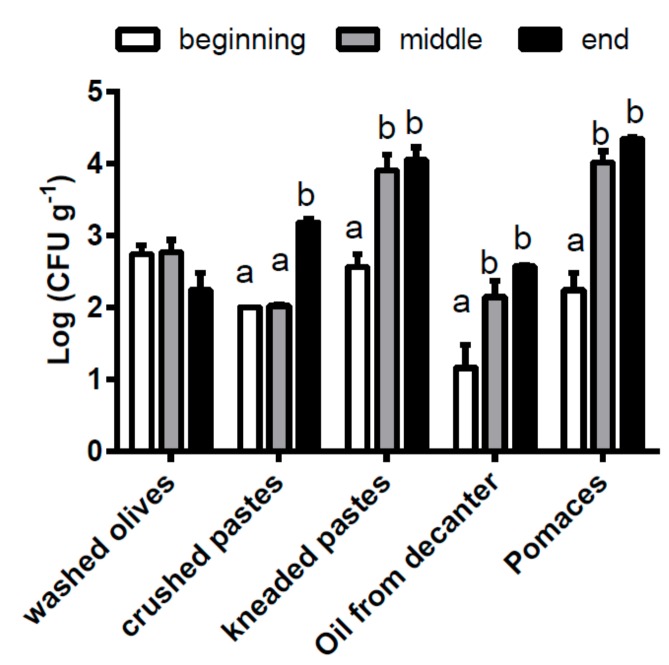
Yeast concentrations at different steps of the oil extraction processes carried out at the beginning (HD1), in the middle (HD2) and the end (HD3) of harvesting in the same crop season. Different letters indicate significant different concentrations within each step (ANOVA, *p* < 0.05).

The yeast populations present in samples of olives as well as of pastes, oil from decanter and pomaces obtained from the extraction processes carried out in the same crop season at the beginning (HD1), in the middle (HD2) and the end (HD3) of harvesting, were quantified (Figure 1). 

The yeast concentration in the olives was not statistically different in the three days of sampling showing an average value of (5.6 ± 1.9) × 10^2^ UFC/g. On the contrary, the yeast concentrations in the kneaded pastes, oil from decanter and pomaces of the first harvesting day (HD1) were statistically lower than the values found in samples from the second and/or the third harvesting day (HD2 and HD3, respectively).

**Figure 2:**
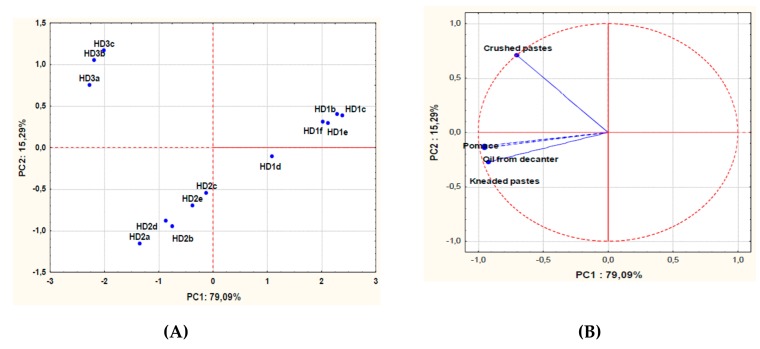
Principal Component Analysis of the yeast concentrations in different samples (pastes, oil from decanter and pomaces) during olive oil extraction processes carried out at the beginning (HD1), in the middle (HD2) and the end (HD3) of harvesting in the same crop season. The scores (**A**) and variable loadings (**B**) for the two first principal components.

A multidimensional map of the yeast concentrations quantified in pastes, oil from decanter and pomaces of the various extraction processes was obtained by PCA. The sample loading and score plots are reported in Figure 2.

The model explained 93% of data variability along the first (PC1) and second (PC2) principal components. The extraction processes clustered together according to the same harvesting date. A comparison between the score plot and the loading plot pointed out that the extraction processes showing a higher contamination by yeasts (HD2 and HD3) were all positioned on the left side of the plot. The extraction processes of the third harvesting date (HD3) were located in the left upper quadrant, crushed pastes being characterized by a higher yeast contamination than in the other processes (HD1 and HD2).

### 3.2. Identification of the Yeast Species

Overall, twelve yeast species belonging to seven genera, besides the yeast-like fungus *Aureobasidium pullulans*, were detected in the different samples collected during the three harvesting days (Table 1). The isolation frequencies of each species were calculated according to the type of sample (olives, pastes, oil, or pomaces) and the harvesting day in which the extraction processes were carried out (Table 1). The comparison among the isolation frequencies highlighted that during the olive oil extractive process some species were typically found in olive fruits whilst other species were associated to crushed and kneaded pastes or found only in oil and in pomaces. 

Moreover, frequencies of yeast species in different samples varied with the harvesting day. Indeed, washed olives were characterized by a significant presence of three different species: *A. pullulans, Candida norvegica*, and *Rhodotorula glutinis* (Table 1) which were often below the detection threshold in other samples. In the crushed pastes *Candida norvegica* and *Rhodotorula mucilaginosa* attained higher percentages than the other species that occurred at frequencies below 1% in samples obtained the first (HD1) and the second (HD2) harvesting day, while in the samples processed on the third harvesting day *A. pullulans* was found at 44%. In the kneaded pastes, the predominant yeast species on the first harvesting day were *Candida kluyveri* and *Saccharomyces cerevisiae* whereas on the second and the third harvesting day *Zygotorulaspora mrakii* and *Nakazawaea wickerhamii*. In oil samples from decanter the yeast species showing significant isolation frequencies were: *Candida adriatica*, *Nakazawaea wickerhamii*, *Nakazawaea molendini-olei*, *Yamadazyma terventina,* and *Metschnikowia fructicola*, although the latter species was isolated only from oil samples of the first harvesting day and *Y. terventina* and *C. adriatica* only from oil samples of the second and third harvesting day. Finally, pomaces were characterized by the presence of the same yeast species isolated from oil samples with the exception of *Z. mrakii*, a species isolated mainly from kneaded pastes of the second and third harvesting day.

### 3.3. Chemical and Sensory Characteristics of Olive Oil Samples

**Figure 3:**
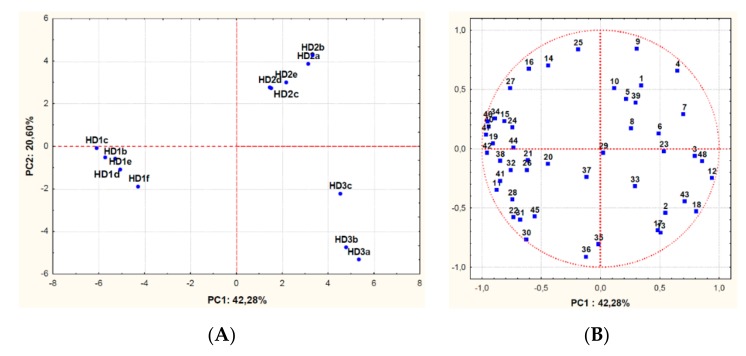
Principal Component Analysis carried out on volatile compounds content of olive oil samples produced during different extractive processes (a, b, c, d, f) at the beginning (HD1), in the middle (HD2) and at the end (HD3) of the same crop season. The scores (**A**) and variable loadings (**B**) for the two first principal components. Variables: (1) Heptane; (2) Octane; (3) Methyl acetate; (4) Ethyl acetate; (5) 2-Butanone; (6) 2-Methyl-butanal; (7) Isovaleraldehydes; (8) Valeraldehydes; (9) Ethyl-vinyl-ketone; (10) Propanol; (11) Hexanal; (12) Isobutanol; (13) 2-Pentanol; (14) trans-2-Pentenal; (15) cis-3-Hexenal; (16) 1-Penten-3-ol; (17) 2-Heptanone; (18) 2 and 3-Methylbutan-1-ol; (19) trans-2-Hexenal; (20) Ocimene; (21) Pentanol; (22) Hexyl acetate; (23) 2-Octanone; (24) Octanal; (25) trans-2-Pentenol; (26) cis-3-Hexenyl acetate; (27) cis-2-Pentenol; (28) trans-2- Hexenyl acetate; (29) 6-Methyl-5-epten-2-one; (30) Hexanol; (31) trans-3-Hexen-1-ol; (32) cis-3-Hexenol; (33) Nonanal; (34) 2,4-Exadienal; (35) trans-2-Hexenol; (36) cis-2-Exenol; (37) trans-2-Octanal; (38) 1-Octen-3-ol; (39) 2,4-Heptadienal; (40) Benzaldehyde; (41) Octanol; (42) Butyric acid; (43) trans-2-Decenal; (44) Nonanol; (45) Ethylbenzene (46) Phenol; (47) 4-Ethylphenol; (48) l-Penten-3-one.

Chemical and sensory analyses of olive oil samples obtained from different extractive processes were performed for oil quality assessment (Table S1 and Table S2). The concentrations of 48 volatile compounds in the 14 olive oil samples were quantified and used to obtain a multidimensional map by PCA, with the exception of data related to oil obtained from the first extractive process (HD1a). Indeed, the plant usually works about three months a year and, therefore, data obtained from the first extractive process might be affected by the environmental conditions occurred during the stopping time and, thus, not be representative. The relevant sample loading and score plots are reported in Figure 3.

The model explained 63% of data variability along the first (PC1) and second (PC2) principal components. All the assayed oil samples clustered according to the harvesting date of the olives. The oils of the first harvesting date were significant different respect to the other oils, being characterized by high values of: 1-penten-3-ol, cis-3-hexenal, cis-3-hexenyl acetate, cis-2-penten-1-ol, trans-2-hexenyl acetate. On the contrary, the most olive oil samples extracted from olives of the second harvesting date contained high concentrations of ethyl vinyl ketone, 2-butanone, propanol, heptane and 2,4-heptadienal. Finally, the oil samples of third harvesting date were characterized by high values of methyl acetate, isobutanol, 2 and 3-methylbutan-1-ol, trans-2-decenal, octane, 2-heptanone, 2-pentanol. The olive oil samples obtained from olives of the first harvesting date (HD1b, HD1c, HD1d, HD1e, and HD1f) and by processes with the lowest level of yeast contamination (Figure 2), grouped together on the left side of the plot (Figure 3). In contrast, the oil samples produced in the middle and at the end of harvesting were positioned on the right side of the plot.

In summary, as the olive harvest proceeded, the oil flavour changed, going e.g. from grassy to more buttery notes as it was shown in Figure 3 considering H1 and H3 samples.

In order to investigate on the possible relation between yeast concentrations found in kneaded pastes or in oil from decanter and the concentrations of volatile compounds in olive oils, correlation studies were carried out and the results are reported in Table 2.

### 3.4. Enzymatic Activity of Yeasts

Yeasts belonging to the species most frequently isolated from decanter oil samples (*C. adriatica*, *N. wickeramii*, *N. molendini-olei*, *Y. terventina*) and coming from different extraction processes were assayed for their enzymatic capabilities with the aim to verify if they could potentially influence the chemical composition of the olive oil. The results are shown in Table 3. All the isolates displayed peroxidase activity, on the contrary no isolates showed cellulase or polygalacturonase activity. All the isolates belonging to *N. molendini-olei* species showed high ß-glucosidase activity, while in the other species this enzymatic activity resulted strain-dependent. Lipase activity was absent in all the isolates of *N. molendini-olei* and present in only one isolate of *N. wickerhamii* species. On the contrary, all isolates of *C. adriatica* and *Y. terventina* species displayed lipase activity. In particular, almost 50% of *Y. terventina* isolates showed high levels of this enzymatic activity.

### 3.5. Yeast Inoculation and Chemical Composition of Extra Virgin Olive Oil (EVOO)

Three strains, belonging to the species most frequently isolated from the extractive processes and detected also in extra virgin olive oil during conservation (*N. molendini-olei* PG194, *N. wickerhamii* DM15, and *Y. terventina* DFX3), were chosen to test whether their enzymatic activities were displayed in EVOO. The three yeast strains showed peroxidase activity, high ß-glucosidase activity and only two of them also high lipase activity (*N. wickerhamii* DM15 and *Y. terventina* DFX3). Isolates of *C. adriatica* were not considered because this species was not frequently found in Tuscan olive oil during conservation. The oil used in the trials was a filtered five-month-old extra virgin olive oil showing a yeast concentration below 10 CFU/mL. To maximize the yeast effect of each species, the three yeast strains were separately inoculated in oil to obtain a final concentration of 10^6^ CFU/mL. During storage, the yeast cells viability in the olive oil decreased according to the isolate inoculated. In detail, the suspended living cells recovered from the samples after two months of storage varied from a minimum of 10^2^ CFU/mL, observed in the olive oil inoculated with *N. molendini-olei*, to a maximum of 10^3^ CFU/mL found in the oil samples inoculated with *N. wickerhamii* and *Y. terventina*. The analytical indices of treated olive oil evaluated after two months of storage, showed some statistical different results for free fatty acids (% oleic acid), peroxide value and total polyphenols (ANOVA, *p* < 0.05). More specifically, the free fatty acids of inoculated oils with *N. wickerhamii* and *Y. terventina* reached values (both 0.28 ± 0.02 % of oleic acid) significantly higher than the control (un-inoculated oil) and the oil inoculated with *N. molendini-olei* (both 0.25 ± 0.01 % of oleic acid). The peroxide values were higher than the control (13.74 ± 0.38 meq O_2_/Kg) only in the olive oil inoculated with *Y. terventina* (16.45 ± 1.41 meq O_2_/Kg). Finally, total polyphenols were 10% lower than that in the control (650 ± 35 mg/kg). In any case, all the inoculated olive oils retained the requirements of extra virgin oil.

**Figure 4:**
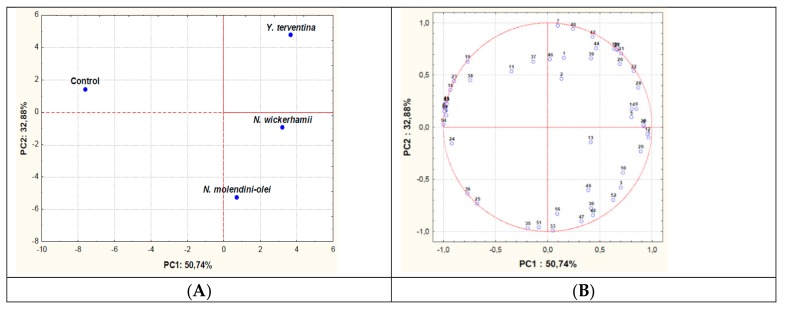
Principal Component Analysis carried out on volatile compounds content of oils inoculated or not (control) with different yeast isolates. The scores (A) and variable loadings (B) for the two first principal components. Variables: (1) l-Octen-3-one; (2) 1-Penten-3-ol; (3) 2,4-Decadienal; (4) 2,4-Heptadienal; (5) trans, trans-2,4-Nonadienal; (6) Butan-2-one; (7) 2 and 3-Methylbutan-1-ol; (8) 2-Methylbutanal; (9) Octan-2-one; (10) l-Penten-3-one; (11) 4-Ethylphenol; (12) 6-Methyl-5-hepten-2-one; (13) trans-2-Decenal; (14) trans-2-Heptenal; (15) trans-2-Hexenal; (16) trans-2-Hexenyl acetate; (17) trans-2-Hexen-1-ol; (18) trans-2-Pentenal; (19) trans-2-Pentenol; (20) trans-2-Hexen-1-ol; (21) Phenol; (22) cis-2-Pentenol; (23) cis-3-Hexenal; (24) cis-3-Hexenyl acetate; (25) cis-3-Hexenol; (26) Butyric acid; (27) Heptanoic acid; (28) Octanoic acid; (29) Pentanoic acid; (30) Propanoic acid; (31) Heptanal; (32) Heptane; (33) Heptan-2-ol; (34) Hexanal; (35) Hexanol; (36) Hexyl acetate; (37) Ethyl acetate; (38) Ethyl isobutyrate; (39) Ethylguaiacol; (40) Ethyl propionate; (41) Ethyl vinyl ketone; (42) Phenylethanol; (43) Guaiacol; (44) Isobutanol; (45) Isovaleraldehydes; (46) Methyl acetate; (47) Ethyl propionate; (48) Nonanol; (49) Octanal; (50) Octane; (51) Octan-2-ol; (52) Pentanol; (53) Propanol; (54) Valeraldehydes.

Volatile compounds content of the control and the inoculated oils were quantified and used to obtain a multidimensional map by PCA. The relevant sample loading and score plots are reported in Figure 4. The model explained 83% of data variability along the first (PC1) and second (PC2) principal components. 

A comparison between the score plot and the loading plot showed that the control was significant different respect to the inoculated oils, which were all positioned on the right side of the plot. Significant differences were also observed between oils inoculated with different yeasts isolates, in particular between *Y. terventina* DFX3 and *N. molendini-olei* PG194.

## 4. Discussion

The yeast concentrations occurring in olive oil extraction processes significantly increased from the first to the last harvesting day of the same crop season. Moreover, the occurrence of different yeast species according to the date of sampling (beginning, middle, and end) demonstrated the progressive contamination of the extraction plant that selects some yeast species at the expense of others. This is the case of some yeast species (as *Z. mrakii* in kneaded pastes as well as *Y. terventina* and *C. adriatica* in oil from decanter) that, being below the detection threshold in the first harvesting date, were then detected at significant level in the second and third harvesting date. The yeast species isolated were in agreement with the results obtained from other surveys carried out on oleic ecosystem [13,16,17,21,27]. Despite all the oil samples were classified as extra virgin olive oils (EU Reg. 1348/2013) [32]., the level of yeast-contamination of the various processes seemed to affect the olive oil chemical composition. Other Authors [17,20,22,23,36] found that some yeast species affect the organoleptic properties of virgin olive oil but no relationships with yeast population concentrations were detected. The oils obtained from processes characterized by a lower yeast contamination (first harvesting date), were characterized by higher concentrations of compounds mostly related to olive oil positive attribute such as “fruity” [1,37,38]. Among these compounds are included cis-3-hexenal and cis-3-hexenyl acetate that are associated with sensory descriptor “Green” [37,38], while cis-2-penten-1-ol to “banana” [37].

On the contrary, the oils obtained from processes more contaminated by yeasts (especially from the third harvesting date) contained higher concentrations of molecules, which were often related to negative attribute [1,3]. Among these compounds were included 2 and 3-methylbutan-1-ol and methyl acetate, 2 and 3-methylbutan-1-ol that are associated with sensory descriptor “winey” and “woody” respectively and both involved in “Mustiness-humidity”, “Fusty” and “Winey-vinegary” negative attribute [3]. Methyl acetate is related to “Winey-vinegary or acid-sour” defect, while trans-2-decenal (sensory descriptor: “painty”, “fishy”, “fatty”) to “Rancid” [1,37,38].

Finally, correlation studies between yeast concentrations in kneaded pastes or oil from decanter and the volatile compounds of the final oils demonstrated significant positive correlations with compounds (i.e. methyl acetate, ethyl acetate, 2 and 3-methylbutan-1-ol) related to “Winey-vinegary or acid-sour” defects. At the same time, significant negative correlations with compounds related to positive attribute were observed (i.e. hexyl acetate; cis-3-hexenyl acetate; trans-2-hexenyl acetate; cis-3- hexenol; 2,4-hexadienal) [1]. In other words, the greater is the yeasts contamination occurring in olive oil extraction processes and the worse is the organoleptic quality of the oil. The only exception was represented by butyric acid, usually related to “rancid” defects [1,3] that was negatively correlated with the yeasts concentration both in kneaded pastes and oil from decanter.

Olive oil chemical characteristics may be affected in different way depending on the enzymatic capabilities of the yeast microbiota occurring in olive oil extraction processes. In fact, most of the enzymatic activities able to modify the olive oil chemical composition were species or strain-dependent as generally reported [1,15,16,24,39].

In this study, peroxidase activity, responsible of a negative influence on olive oil quality due to oxidative degradation of the protective phenol compounds [40] was common to all the species assayed. On the contrary, cellulase and polygalacturonase activities that increase antioxidant phenol compound levels conferring a protective effect by hydrolysing olive cell-wall polysaccharides [41], were absent in all the assayed isolates. Finally, β-glucosidase and lipase activities were strain and/or species-dependent. The β-glucosidase enzyme is involved in the degradation of oleuropeine into a heterosidic ester of elenolic acid and 3,4-dihydroxyphenylethanol; both of these compounds are technologically important in view of their browning capacity and intense bitter taste [11,12,42].

Lipase activity can impair product quality due to the increase of both the diglyceride and acidity levels through hydrolysis of triacylglycerols [24,43]. Considering that the olives are fruits with high fat concentrations, the presence of lipolytic yeasts in olive oil could modify the nutritional composition and organoleptic characteristic of this product.

When three representative strains (*N. molendini-olei* PG194, *N. wickerhamii* DM15, *Y. terventina* DFX3) characterized by different enzymatic capabilities were inoculated in olive oil, different effects on oil chemical composition were detected. The analytical indices, used to classify an olive oil as extra-virgin, showed significant differences: the acidity level increased when *C. wickeramii* DM15 and *Y. terventina* DFX3 were present; peroxide values increased only in the presence of *Y. terventina* DFX3, total polyphenols decreased independently of the inoculated yeast strain. 

Finally, also the volatile compounds content that resulted were strongly influenced by the yeast strain inoculated. 

To generalize, in the samples of oil treated with yeasts, a higher concentration of some compounds responsible of negative oil attributes (i.e.: trans 2-heptenal, 6-methyl-5-hepten-2-one, 2-octanone) and a lower concentration of C6 volatile carbonyl compounds responsible for positive oil attributes, were found. Similarly, Zullo et al. [21] observed a lower content of C6 volatile carbonyl compounds when a *N. wickerhamii* strain was inoculated in oil.

## 5. Conclusions

In conclusion, during the olive oil crop season, some yeast species colonize the extraction plant (malaxation equipment and decanter in particular) at the expense of others becoming the dominant microbiota. This colonization significantly affects the volatile compound content of the olive oils; indeed, the oils obtained in the first days of the olive oil crop season were significantly different from the others. The effects of the yeasts colonization on the chemical characteristics of the oils depend on not only by the population density but also by the enzymatic capabilities of the species and/or the strains composing the microbiota. Therefore, the hygienic condition of the olive oil extraction plant is important in the definition of an olive oil aromatic profile. In this contest, it could be of interest to investigate if each olive oil extraction plant might select a typical microbiota, with metabolic capabilities potentially able to affect in a characteristic way the aromatic composition of the final product.

## Figures and Tables

**Table 1:** Isolation frequencies (%) of yeast species and of the yeast-like fungus *A. pullulans* in different samples collected during olive oil extraction processes carried out at the beginning (HD1), in the middle (HD2), and at the end (HD3) of the same crop season (the symbol “-” indicates isolation frequency < 1%).

Yeast species	Washed Olives			Crushed Pastes			Kneaded Pastes			Oil from Decanter			Pomaces		
	HD1	HD2	HD3	HD1	HD2	HD3	HD1	HD2	HD3	HD1	HD2	HD3	HD1	HD2	HD3
*Aureobasidium pullulans*	83	100	25	-	-	44	17	-	-	-	-	-	-	-	-
*Candida adriatica*	-	-	-	-	-	-	-	-	-	-	25	29	-	40	9
*Candida diddensiae*	-	-	-	-	-	-	6	-	-	-	-	-	-	-	-
*Candida kluyveri*	-	-	-	-	-	-	53	-	-	-	-	-	-	-	-
*Candida norvegica*	12	-	50	50	33	13	-	-	-	-	-	-	-	-	-
*Nakazawaea wickerhamii*	-	-	-	-	-	5	-	4	26	62	24	4	-	9	14
*Nakazawaea molendini-olei*	-	-	-	-	-	5	6	4	13	13	16	4	-	11	11
*Metschnikowia fructicola*	-	-	-	-	-	5	-	-	-	25	-	-	-	-	-
*Rhodotorula glutinis*	-	-	25	-	-	9	-	-	-	-	-	-	-	-	-
*Rhodotorula mucilaginosa*	-	-	-	50	77	-	-	-	-	-	-	-	11	-	-
*Saccharomyces cerevisiae*	-	-	-	-	-	-	18	-	8	-	-	-	-	-	-
*Yamadazyma terventina*	-	-	-	-	-	-	-	-	-	-	35	59	-	5	16
*Zygotorulaspora mrakii*	-	-	-	-	-	-	-	87	53	-	-	4	55	31	50
Others	5	-	-	-	-	19	-	5	-	-	-	-	34	4	-

**Table 2:** Statistically significant correlations (*p* < 0.05) calculated between yeast concentrations occurring in kneaded pastes or oil from decanter and volatile compounds of the final olive oil samples. (ns = not significant).

Compounds	Kneaded Pastes		Oil from Decanter	
	Spearman r	Pearson r	Spearman r	Pearson r
*Aldehydes*				
Hexanal	−0.6998	ns	−0.7660	−0.6321
cis-3-Hexenal	−0.6203	−0.633	−0.7660	−0.8160
trans-2-Hexenal	−0.5982	−0.603	−0.6115	−0.5791
2-Methyl-butanal	0.6556	ns	0.6733	0.6797
Isovaleraldehydes	0.9161	0.7170	0.8808	0.8371
2,4-Heptadienal	−0.7086	−0.6413	−0.7704	−0.7863
Benzaldehydes	−0.7572	ns	−0.7616	−0.5704
trans-2-Decenal	0.5378	0.5590	ns	ns
*Esters*				
Methyl acetate	0.8013	0.7186	0.8013	0.7650
Ethyl acetate	0.7572	0.6547	0.7130	0.5935
Hexyl acetate	−0.7925	−0.6573	−0.8499	−0.8145
trans-2- Hexenyl acetate	−0.7339	−0.8582	−0.7251	−0.7072
cis-3-Hexenyl acetate	−0.7484	−0.6344	−0.6954	−0.8006
4-Ethyl-phenol	−0.7042	−0.5673	−0.7439	−0.6407
*Carboxylic acids and ketones*				
Butyric acid	−0.6733	−0.6317	−0,691	−0.6885
l-Penten-3-one	0.8102	0.7454	0.713	0.7172
*Alcohols*				
Isobutanol	0.8318	0.7596	0.7628	0.7562
2 and 3-Methylbutan-1-ol	0.8013	0.6240	0.7704	0.6024
trans-3-Hexen-1-ol	−0.7307	−0.6868	−0.7042	−0.7060
cis-3-Hexenol	−0.7660	−0.6455	−0.7881	−0.8419
Nonanol	−0.5938	−0.4505	−0.5717	ns
Hexanol	−0.7484	−0.6560	−0.6954	−0.6181
1-Octen-3-ol	−0.6556	ns	−0.6998	−0.5398

**Table 3:** Enzymatic activities of the yeast species isolated from olive oil samples obtained from decanter in different extraction processes.

Yeast Species	N. of Isolates	Enzymatic Activity									
		ß-glucosidase				Lipase				Peroxidase	
		**-**	**+**	++	+++	-	+	++	+++	-	+
*C. adriatica*	13	0	1	3	9	0	4	8	1	0	13
*N. molendini-olei*	23	0	0	0	23	23	0	0	0	0	23
*N. wickerhamii*	21	4	0	4	13	20	0	0	1	0	21
*Y. terventina*	15	6	5	3	1	0	6	1	8	0	15

## Supplementary Materials

The following are available online at https://www.mdpi.com/2304-8158/8/10/457/s1, Table S1: Chemical analyses of olive oil samples obtained from different extractive processes (a, b, c, d, f) carried out at the beginning (HD1), in the middle (HD2) and the end (HD3) of harvesting in the same crop season (U = measurement uncertainty), Table S2: Volatile compounds (mg/kg) in olive oil samples obtained from different extractive processes (a, b, c, d, f) carried out at the beginning (HD1), in the middle (HD2) and the end (HD3) of harvesting in the same crop season.
