# Supplementary material for: Extra Virgin Olive Oil Quality as Affected by Yeast Species Occurring in the Extraction Process
Source: Foods. 2019 Oct 7;8(10):457. doi: 10.3390/foods8100457 (PMC6835964; doi:10.3390/foods8100457)
Supplement: Supplementary file 1 [file foods-08-00457-s001.pdf]

Table S1. Chemical analyses of olive oil samples obtained from different extractive processes (a, b, c, d, f) carried out at the beginning (HD1), in the middle (HD2) and the end (HD3) of harvesting in the same crop season (U = measurement uncertainty)

	Acidity (Percentage of Oleic Acid)	U	Peroxide Value (meq O_2/Kg)	U	Total Phenolic Concentration (mg/Kg of Gallic Acid)	U
**HD1a**	0.23	± 0.01	2.3	± 0.80	521	± 55
**HD1b**	0.26	± 0.01	1.4	± 0.80	654	± 72
**HD1c**	0.31	± 0.01	1.6	± 0.80	771	± 92
**HD1d**	0.26	± 0.01	1.6	± 0.80	656	± 73
**HD1e**	0.25	± 0.01	2.1	± 0.80	598	±65
**HD1f**	0.31	± 0.01	2.1	± 0.80	712	± 81
**HD2a**	0.28	± 0.01	3.1	± 0.80	746	± 87
**HD2b**	0.24	± 0.01	2.6	± 0.80	657	± 73
**HD2c**	0.28	± 0.01	2.7	± 0.80	663	± 74
**HD2d**	0.28	± 0.01	2.3	± 0.80	591	± 64
**HD2e**	0.23	± 0.01	2.5	± 0.80	699	± 79
**HD3a**	0.27	± 0.01	4.5	± 0.80	569	± 61
**HD3b**	0.26	± 0.01	4.4	± 0.80	573	± 62
**HD3c**	0.25	± 0.01	3.2	± 0.80	636	± 70

Table 2. Volatile compounds (mg/kg) in olive oil samples obtained from different extractive processes (a, b, c, d, f) carried out at the beginning (HD1), in the middle (HD2) and the end (HD3) of harvesting in the same crop season.

(1) Heptane; (2) Octane; (3) Methyl acetate; (4) Ethyl acetate; (5) 2-Butanone; (6) 2-Methyl-butanal; (7) Isovaleraldehydes; (8) Valeraldehydes; (9) Ethyl-vinyl-ketone; (10) Propanol; (11) Hexanal; (12) Isobutanol; (13) 2-Pentanol; (14) trans-2-Pentenal; (15) cis-3-Hexenal; (16) 1-Penten-3-ol; (17) 2-Heptanone; (18) 2 and 3-Methylbutan-1-ol; (19) trans-2-Hexenal; (20) Ocimene; (21) Pentanol; (22) Hexyl acetate; (23) 2-Octanone; (24) Octanal; (25) trans-2-Pentenol; (26) cis-3-Hexenyl acetate; (27) cis-2-Pentenol; (28) trans-2- Hexenyl acetate; (29) 6-Methyl-5-epten-2-one; (30) Hexanol; (31) trans-3-Hexen-1-ol; (32) cis-3-Hexenol; (33) Nonanal; (34) 2,4-Exadienal; (35) trans-2-Hexenol; (36) cis-2-Exenol; (37) trans-2-Octanal; (38) 1-Octen-3-ol; (39) 2,4-Heptadienal; (40) Benzaldehydes; (41) Octanol; (42) Butyric acid; (43) trans-2-Decenal; (44) Nonanol; (45) Ethylbenzene (46) Phenol; (47) 4-Ethylphenol; (48) l-Penten-3-one.

	1	2	2	4	5	6	7	8	9	10	11	12	13	14	15	16
**HD1a**	0.009	0.029	0.004	0.001	0.017	0.033	0.007	0.060	0.394	0.003	1.036	0.000	0.000	0.078	5.958	0.359
**HD1b**	0.004	0.014	0.001	0.000	0.001	0.023	0.004	0.034	0.459	0.002	0.816	0.000	0.000	0.072	6.621	0.432
**HD1c**	0.002	0.013	0.002	0.000	0.001	0.027	0.005	0.041	0.603	0.002	0.790	0.000	0.000	0.082	6.305	0.549
**HD1d**	0.003	0.014	0.002	0.000	0.001	0.029	0.004	0.044	0.547	0.002	0.719	0.000	0.000	0.077	5.978	0.528
**HD1e**	0.002	0.011	0.001	0.000	0.001	0.018	0.003	0.035	0.481	0.002	0.578	0.000	0.000	0.071	7.004	0.515
**HD1f**	0.002	0.012	0.001	0.000	0.001	0.019	0.003	0.040	0.463	0.001	0.572	0.000	0.000	0.063	5.652	0.497
**HD2a**	0.004	0.023	0.005	0.011	0.009	0.036	0.012	0.074	0.679	0.002	0.272	0.004	0.000	0.069	1.793	0.443
**HD2b**	0.004	0.019	0.006	0.016	0.002	0.046	0.017	0.067	0.791	0.002	0.276	0.006	0.000	0.077	1.820	0.533
**HD2c**	0.003	0.015	0.008	0.017	0.001	0.027	0.007	0.028	0.725	0.003	0.350	0.004	0.000	0.081	5.692	0.520
**HD2d**	0.003	0.011	0.006	0.012	0.001	0.019	0.006	0.022	0.760	0.002	0.312	0.005	0.000	0.082	5.693	0.517
**HD2e**	0.003	0.011	0.006	0.017	0.001	0.018	0.005	0.031	0.642	0.002	0.317	0.006	0.000	0.076	6.405	0.489
**HD3a**	0.003	0.057	0.011	0.006	0.001	0.034	0.009	0.044	0.513	0.002	0.440	0.011	0.004	0.060	1.591	0.340
**HD3b**	0.003	0.032	0.007	0.007	0.001	0.030	0.009	0.048	0.477	0.002	0.401	0.009	0.002	0.055	1.439	0.329
**HD3c**	0.002	0.014	0.003	0.004	0.001	0.029	0.008	0.037	0.479	0.002	0.303	0.008	0.000	0.049	1.326	0.341

	17	18	19	20	21	22	23	24	25	26	27	28	29	30	31	32
**HD1a**	0.002	0.009	13.781	0.000	0.011	0.174	0.020	0.066	0.054	0.888	0.529	0.000	0.015	0.634	0.017	1.441
**HD1b**	0.000	0.006	12.826	0.000	0.005	0.210	0.000	0.025	0.056	0.997	0.526	0.061	0.009	0.587	0.013	1.281
**HD1c**	0.000	0.008	12.635	0.129	0.006	0.208	0.000	0.027	0.066	1.193	0.614	0.057	0.010	0.599	0.016	1.411
**HD1d**	0.000	0.006	12.068	0.104	0.006	0.182	0.000	0.028	0.061	1.152	0.604	0.064	0.004	0.583	0.016	1.382
**HD1e**	0.000	0.007	10.299	0.000	0.004	0.187	0.000	0.021	0.061	1.363	0.522	0.132	0.005	0.604	0.015	1.317
**HD1f**	0.000	0.006	11.665	0.000	0.003	0.170	0.000	0.023	0.058	1.238	0.505	0.113	0.007	0.647	0.017	1.364
**HD2a**	0.000	0.011	8.884	0.000	0.004	0.053	0.000	0.024	0.065	0.277	0.435	0.000	0.006	0.257	0.004	0.434
**HD2b**	0.000	0.017	9.666	0.000	0.004	0.037	0.000	0.022	0.081	0.275	0.528	0.000	0.004	0.280	0.005	0.534
**HD2c**	0.000	0.014	8.069	0.022	0.004	0.105	0.005	0.000	0.064	1.155	0.537	0.019	0.010	0.367	0.012	1.203
**HD2d**	0.000	0.012	7.322	0.023	0.004	0.109	0.005	0.000	0.067	1.306	0.523	0.024	0.009	0.353	0.011	1.329

**HD2e**	0.000	0.015	7.600	0.000	0.003	0.103	0.006	0.000	0.062	1.072	0.528	0.016	0.009	0.273	0.009	1.132
**HD3a**	0.000	0.035	7.527	0.026	0.004	0.131	0.003	0.000	0.048	0.734	0.372	0.024	0.009	0.547	0.012	0.878
**HD3b**	0.000	0.038	6.942	0.015	0.004	0.134	0.002	0.000	0.048	0.763	0.368	0.038	0.007	0.546	0.013	0.931
**HD3c**	0.000	0.026	6.452	0.024	0.003	0.158	0.011	0.000	0.044	0.846	0.351	0.033	0.007	0.449	0.009	0.773

	**33**	**34**	**35**	**36**	**37**	**38**	**39**	**40**	**41**	**42**	**43**	**44**	**45**	**46**	**47**	**48**
**HD1a**	0.583	1.623	0.462	0.009	0.065	0.010	0.000	0.094	0.097	0.201	0.000	0.104	0.294	0.984	0.098	0.015
**HD1b**	0.282	1.507	0.363	0.004	0.035	0.005	0.000	0.058	0.048	0.182	0.000	0.097	0.201	0.361	0.069	0.020
**HD1c**	0.229	1.448	0.375	0.005	0.022	0.007	0.000	0.072	0.046	0.155	0.000	0.063	0.201	0.353	0.063	0.030
**HD1d**	0.217	1.419	0.403	0.005	0.034	0.008	0.000	0.066	0.052	0.118	0.000	0.060	0.193	0.350	0.057	0.025
**HD1e**	0.249	1.484	0.521	0.004	0.022	0.005	0.000	0.058	0.046	0.134	0.000	0.071	0.159	0.333	0.062	0.018
**HD1f**	0.191	1.285	0.512	0.006	0.020	0.004	0.000	0.051	0.050	0.114	0.000	0.065	0.167	0.321	0.049	0.019
**HD2a**	0.202	0.730	0.311	0.002	0.024	0.003	0.000	0.042	0.035	0.047	0.000	0.056	0.133	0.266	0.033	0.031
**HD2b**	0.171	0.757	0.387	0.003	0.017	0.004	0.000	0.039	0.033	0.024	0.000	0.055	0.144	0.274	0.025	0.040
**HD2c**	0.352	1.216	0.278	0.003	0.022	0.003	0.010	0.045	0.027	0.032	0.091	0.049	0.136	0.260	0.031	0.032
**HD2d**	0.266	1.182	0.188	0.002	0.036	0.003	0.014	0.043	0.027	0.028	0.122	0.026	0.122	0.252	0.027	0.035
**HD2e**	0.322	1.289	0.176	0.002	0.024	0.003	0.010	0.046	0.024	0.012	0.099	0.026	0.098	0.265	0.036	0.035
**HD3a**	0.362	0.594	0.650	0.006	0.030	0.003	0.000	0.032	0.037	0.002	0.234	0.046	0.183	0.203	0.014	0.049
**HD3b**	0.375	0.532	0.586	0.006	0.028	0.004	0.005	0.030	0.029	0.002	0.145	0.029	0.177	0.197	0.015	0.041
**HD3c**	0.168	0.535	0.339	0.005	0.025	0.002	0.000	0.029	0.025	0.002	0.103	0.019	0.128	0.192	0.013	0.035
